# Characterization of the Prognostic m6A-Related lncRNA Signature in Gastric Cancer

**DOI:** 10.3389/fonc.2021.630260

**Published:** 2021-04-13

**Authors:** Haixu Wang, Qingkai Meng, Bin Ma

**Affiliations:** ^1^ Postgraduate Training Base in General Hospital of The Northern Theater Command, China Medical University, Shenyang, China; ^2^ Department of Radiation Oncology, The General Hospital of Northern Theater Command, Shenyang, China; ^3^ Department of Colorectal Surgery, Cancer Hospital of China Medical University, Liaoning Cancer Hospital and Institute, Shenyang, China

**Keywords:** N6-methyladenosine, gastric cancer, lncRNA signature, nomogram, molecular subgroups, immunotherapy

## Abstract

N6-methyladenosine (m^6^A) is a common form of mRNA modification regulated by m6A RNA methylation regulators and play an important role in the progression of gastric cancer (GC). However, the prognostic role of m^6^A-related lncRNA in gastric cancer has not been fully explored. This study aims at exploring the biological function and prognostic roles of the m^6^A-related lncRNA signature in gastric cancer. A total of 800 m6A-related lncRNAs were identified through Pearson correlation analysis between m6A regulators and all lncRNAs. Eleven m6A-related lncRNA signatures were identified through a survival analysis and the Kaplan-Meier (KM) curve analysis results suggest that patients in the low-risk group have a better overall survival (OS) and disease-free survival (DFS) outcome than the high-risk group. Also, the lncRNA signature can serve as an independent prognostic factor for OS and DFS. The gene set enrichment analysis (GSEA) result suggests that patients in the high-risk group were mainly enriched in the ECM receptor interaction, focal adhesion, and cytokine-cytokine receptor interaction pathway, while the low-risk group was characterized by the base excision repair pathway. We further constructed an individualized prognostic prediction model *via* the nomogram based on the independent prognostic factor for the OS and DFS, respectively. In addition, some candidate drugs aimed at GC risk group differentiation were identified using the Connective Map (CMAP) database. Lastly, four subgroups (C1, C2, C3, and C4) were identified based on the m6A-related lncRNA expression, through a consensus clustering algorithm. Among them, C1 and C2 have a greater likelihood to respond to immune checkpoint inhibitor immunotherapy, suggesting that the C1 and C2 subgroup might benefit from immunotherapy. In conclusion, the m6A-related lncRNA signature can independently predict the OS and DFS of GC and may aid in development of personalized immunotherapy strategies.

## Introduction

Gastric carcinoma is one of the most common malignant tumours and the third leading cause of cancer-related mortality (1). It is associated with poor prognosis with about 80% of patients being diagnosed at an advanced stage (2). Surgery is the most effective therapy for gastric cancer. However, full recovery is not guaranteed for patients with recurrent or un-resectable GC. Notably, the 5-year mortality rate for advanced gastric cancer is between 30% to 50% (3). Therefore, there is a need to explore alternative prognostic markers.

Studies report that RNA modification plays a crucial role in the post-transcriptional regulation of gene expression (4). A total of 163 different RNA modifications were reported in all living organisms (5). N6-methyladenosine (m6A) modifications are the most common RNA modifications and have been widely studied. m6A methylation is a dynamic reversible process carried out by methyltransferase complex (writers), demethylase (erasers), and function manager (readers) (6). The methyltransferase complex is composed of METTL3, METTL14, KIAA1429, WTAP, RBM15, and ZC3H13 which mediate the RNA methylation modification process (7). Demethylase is composed of FTO and ALKBH5, and mediates the RNA demethylation process (8, 9). On the other hand, the function manager includes YTHDC1, YTHDC2, YTHDF1, YTHDF2,and HNRNPC and play a role in “reading” RNA methylated information, and the translation and degradation of downstream RNA (10). m6A through the “writers” link methyl groups to RNA which is further recognized by the “readers” to aid processes such as RNA processing, nuclear export, translation, and decay. Further, m6A plays a role in gene expression regulation through demethylation of RNA by demethylase (11).

Although the human genome can transcribe nearly 60,000 genes, about 20,000 genes are protein coding genes, and the remaining genes mainly belong to non-coding genes. Among them, about 16,000 genes were lncRNAs which account for a quarter of the total genes. lncRNA are at least 200 bp long and have a lower protein-coding potential compared to miRNA and snRNA (12). Previous studies report that lncRNAs play an important role in transcriptional and post transcriptional regulation and chromatin modification through the regulation of gene expression (13). Recent studies have reported aberrant lncRNAs expression as diagnostic and prognostic markers in tumors (14). However, the role of lncRNAs in m6A modification in GC have not been reported.

In the present study, we analyzed the role of m6A-related lncRNAs in overall survival of GC patients. We constructed 11 m6A-related lncRNA signatures and further validated this in the testing dataset, complete dataset, and DFS dataset, respectively. Notably, the lncRNA signature can be used as an independent prognostic marker for GC without the need to consider other clinical variables. In addition, four subgroups with distinct benefits to immunotherapy were identified using a non-negative matrix factorization (NMF) analysis, based on the expression of lncRNA.

## Materials and Methods

### Data Collection and Correlation Analysis

The RNA transcriptome dataset and the corresponding GC clinical information were downloaded from The Cancer Genome Atlas (TCGA, https://portal.gdc.cancer.gov/) database. Genes were grouped into protein coding genes and lncRNA genes based on the human genome annotation data. Expression levels of 13 m6A regulator genes were also determined. We used the Pearson correlation coefficient to evaluate the correlation between m6A regulator genes and lncRNA. The lncRNA with an absolute correlation coefficient >0.3 and a P value <0.05 were considered as a m6A-related lncRNA. Patients were then grouped into two groups: the training dataset and the testing dataset. Retrieved data were used for subsequent bioinformatics analysis.

### Risk Model Construction

Univariate cox regression analysis and least absolute shrinkage and selection operator (LASSO)-penalized Cox regression analysis were employed to identify the m6A-related lncRNA prognostic signature in the training dataset. The risk score for each GC patient was calculated based on the following formula:

Risk score = Σ_Expi *βi_. where Exp_i_ represents each lncRNA expression and β_i_ represents the coefficient of each lncRNA. The receiver operating characteristic (ROC) curve analysis was used to evaluate the accuracy of the lncRNA signature in the training dataset, testing dataset, the complete dataset, and the DFS dataset.

### Relationship Between the lncRNA Signature and Clinical Parameters in GC

The complete dataset and DFS dataset with corresponding clinical information were used for subsequent analysis. To identify the independence of the lncRNA signature, we performed a univariate cox regression and multivariate cox regression analysis between the lncRNA signature and the clinical traits, and the significant independent prognostic factors was selected based on a *P* value <0.05.

### Construction and Validation of Nomogram

The nomogram prediction model was constructed based on the lncRNA signature risk score and independent clinical factors, using the “rms” R package. The calibration plot was applied to validate the calibration and accuracy of the nomogram (by a bootstrap method with 1,000 replicates).

### Gene Set Enrichment Analysis

The Gene Set Enrichment Analyses (GSEA) method was used to explore the potential KEGG pathway implicated in the lncRNA prognostic signature. The reference gene set was retrieved from c2.cp.kegg.v7.1.symbols files, and the significant pathways were screened based on the criterion: *P <*0.05 and *FDR <*0.25.

### Non−Negative Matrix Factorization Consensus Clustering

We used non-negative matrix factorization (NMF) clustering methods to explore potential molecular subgroups based on the prognostic lncRNA expression profile. The optimal K cluster was selected on the basis of the cophenetic correlation coefficient. We further used the TIDE algorithm to predict the response of the four subgroups to immunotherapy.

### Small Molecular Drug Prediction

The differentially expression genes (DEGs) between the high-risk group and the low-risk group were identified using the “limma” R package. We then uploaded the top 1,000 DEGs to the CMAP database to identify which target compounds might be useful.

### Statistical Analysis

Computational and statistical analyses were performed using R software (https://www.r-project.org/). The Kruskal-Wallis test was used to compare the four subgroups and to explore their response to immunotherapy. Differences between the high-risk group and the low-risk group were determined using the Kaplan-Meier curve and log-rank test. Clinical data were analyzed using the chi-square test or Fisher’s exact test. For all analyses, a *P* value <0.05 was considered statistically significant.

## Results

### Expression Profiles of m6A RNA Methylation Regulators in GC

m6A RNA methylation regulators play a crucial role in progression of malignant tumors; therefore, we explored the expression profiles of 13 m6A RNA methylation regulators in GC. High expression levels of the 13 genes were observed in the tumor tissue compared with normal tissue (**Figure S1A**). Notably, the tumor tissue showed significantly higher expression levels of m6A regulators, except for FTO and ALKBH5, compared with the normal tissue with a *P* value <0.05. Further, the correlation analysis showed that HNRNPC and YDHDF2 methylation regulators were highly correlated with GC progression (Cor = 0.57) (**Figure S1B**).

### Identification of m6A-Related lncRNAs in GC

To identify potential m6A-related lncRNAs, the Pearson correlation coefficient was used to assess the relationship between m6A methylation regulators and lncRNAs. As a result, we identified 1,351 interactions and 800 m6A-related lncRNAs with an absolute correlation coefficient >0.3 and a *P* value <0.05. Among them, 117 interactions were identified between lncRNAs and m6A methylation regulators (**Table S1**).

### lncRNA Signature Construction

**Figure 1:**
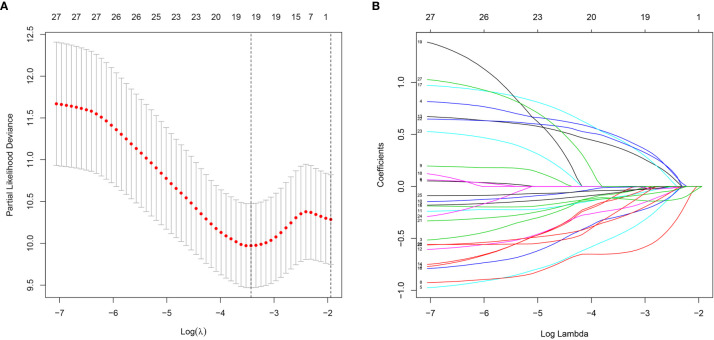
Characterization of m6A-related lncRNAs signature. **(A)** The LASSO coefficient of 13 genes in GC. **(B)** Selecting the best parameters for GC on the basis of LASSO model (λ).

**Figure 2:**
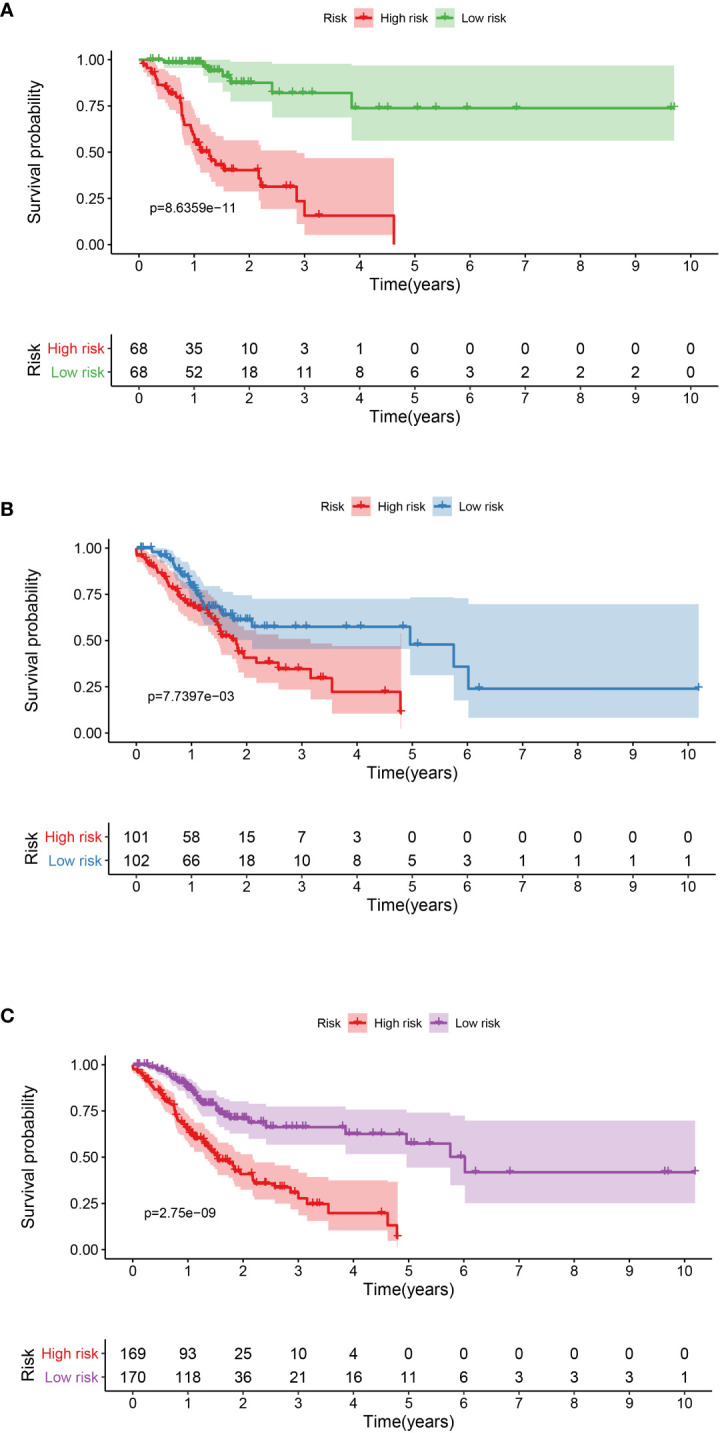
Kaplan-Meier curve analysis between the high-risk group and low-risk group was performed in the training dataset **(A)**, testing dataset **(B)**, and complete dataset **(C)**, respectively.

**Figure 3:**
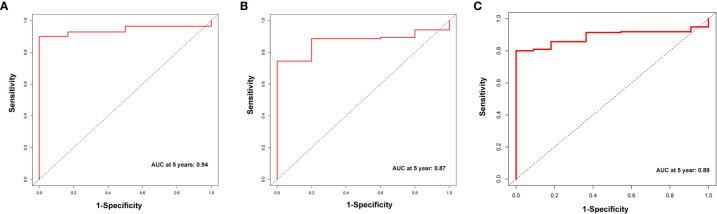
The receiver operator curve (ROC) analysis for the m6A-related lncRNAs signature in the training dataset **(A)**, testing dataset **(B)**, and complete dataset **(C)**, respectively.

A total of 339 patient samples were grouped into a training dataset (N = 136) and a testing dataset (N =203). We then performed a univariate cox regression analysis and LASSO-penalized Cox regression analysis to construct a 11-lncRNA signature model in the training dataset (**Figure 1**). The risk score for each patient in the training dataset, testing dataset, and the complete dataset was calculated based on the risk formula: risk score = AL049840.3 * 0.599866058 + AC008770.3 * (-1.237087957) + AL355312.3 * (-0.19130367) + AC108693.2 * (-0.956067535) + BACE1-AS * (-0.362760192) + AP001528.1 * 0.528553101 + AP001033.2 * 0.594102051 + AC092574.1 * (-0.618599189) + AC010719.1 * (-0.337936563) + AC009090.3 * 0.770122519 + SAMD12-AS1 * (-0.766369919). Patients in the training dataset, testing dataset, and complete dataset were further grouped into a high-risk group and low-risk group based on the median risk score. We found that patients in the high-risk group corresponded to a greater number of deaths than the low-risk group patients in the training dataset and testing dataset, respectively (**Figure S2**). The Kaplan-Meier (KM) curve analysis results showed that the low-risk group have a better prognosis than the high-risk group in the testing, training, and the complete dataset (*P <*0.05) (**Figure 2**). In addition, the area under the curve (AUC) for 5-year overall survival (OS) was 0.94, 0.85, and 0.89 in the training dataset, testing dataset, and complete dataset, respectively, implying that the lncRNA signature has a good accuracy in the prognostic prediction of GC (**Figure 3**).

### Prognostic Value of lncRNA Signature

**Figure 4:**
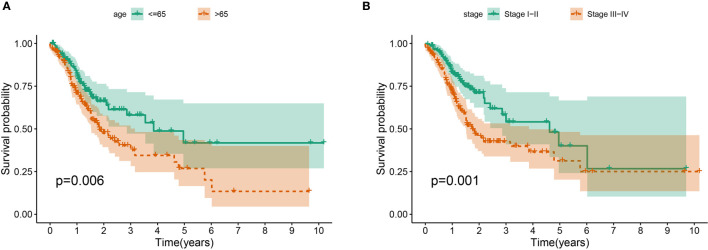
The prognostic value was evaluated based on the age **(A)** and stage **(B)** in the complete dataset.

**Figure 5:**
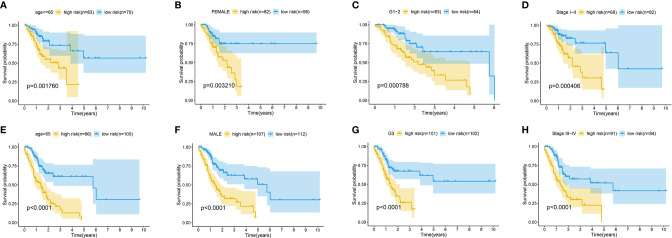
Kaplan-Meier survival curves for the high-risk and low-risk groups stratified by clinical factors including age **(A, E)**, gender **(B, F)**, grade **(C, G)**, and stage **(D, H)** in the complete dataset.

The association between the lncRNA signature and clinical factors were evaluated by performing a KM curves analysis. As shown in **Figure 4A**, we found that the risk score significantly increased from the younger group to older group (*P <*0.05). Moreover, the stage also presented a significant divergence from stage I to stage IV (*P* < 0.05) (**Figure 4B**). These results indicate that the greater the progression is, the higher the risk score of GC is. Moreover, we also investigated the prognostic value of the m6A-reated lncRNA signature in GC patients stratified by clinical pathological variables, including age, gender, cancer grade, and stage. For all different stratifications, patients in the high-risk group tended to have a lower overall survival rate compared to the low-risk group (**Figures 5A–H**). These results suggest that the m6A-related lncRNA signature can predict the prognosis of GC without the need to consider clinical factors.

### Independent Prognostic Role of the lncRNA Signature

**Figure 6:**
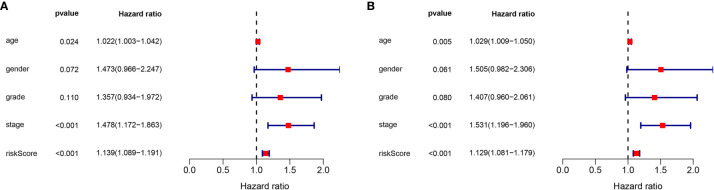
Validation of the independence of the m6A-related lncRNA signature in OS through the Univariate cox regression analysis **(A)** and Multivariate cox regression analysis **(B)**.

To determine the independence of the lncRNA signature in the clinical factors, we conducted a univariate cox regression analysis and multivariate cox regression analysis between the lncRNA signature and the clinical factors in the complete dataset. As shown in **Figures 6A, B**, we found that the lncRNA signature can act as an independent prognostic factor for the prognosis of GC.

### Gene Set Enrichment Analyses (GSEA)

**Figure 7:**
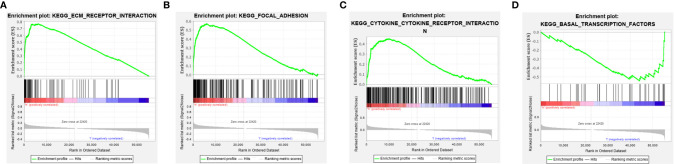
Gene set enrichment analysis (GSEA) of the m6A-related lncRNA signature in the high-risk group **(A–C)** and low-risk group **(D)**, respectively.

The potential pathways or functions of the m6A-related lncRNA signature were explored by performing a Gene Set Enrichment analysis. As shown in **Figures 7A–C**, we discovered that patients in the high-risk group were mainly involved in the cytokine receptor interaction, focal adhesion, and ECM receptor interaction pathway, while basal transcription factors pathway were enriched in the low-risk group (**Figure 7D**).

### Validation of the lncRNA Signature

**Figure 8:**
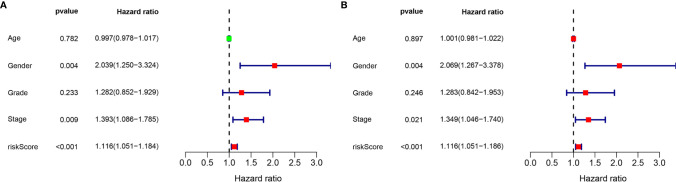
Validation of the independence of the m6A-related lncRNA signature in DFS through the Univariate cox regression analysis **(A)** and Multivariate cox regression analysis **(B)**.

Considering the significance of the disease-free survival in the prognosis of GC, we further calculated the risk score for each GC patient based on the previous risk formula. The KM curve analysis result suggests that patients in the high-risk group have a poor survival compared to the low-risk group (**Figure S3A**). The ROC curve analysis results suggest that the lncRNA signature in the DFS have a good accuracy of prognostic prediction (**Figure S3B**). Moreover, the univariate cox regression and multivariate cox regression analysis suggest that the lncRNA signature can serve as an independent prognostic factor for the prognostic prediction of GC (**Figures 8A, B**). Moreover, we also compared our risk model with other reported lncRNA signatures, such as Wu et al. (15) and Chen et al. (16). As shown in **Figure S4**, we observed that our risk signature has the highest AUC value when compared with other risk models, suggesting that our signature is a reliable prognostic model.

### Individualized Prediction Model Construction

**Figure 9:**
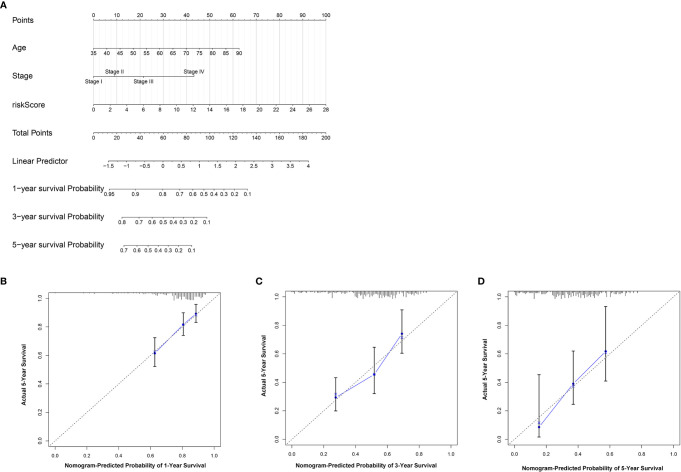
Construction of nomogram to predict the prognostic ability for the OS in the complete dataset based on the independent factors. **(A)** A nomogram was constructed based on the independent prognosis factors to predict the prognosis of GC. The calibration plots for 1- **(B)**, 3- **(C)** and 5-years **(D)** in the complete dataset.

**Figure 10:**
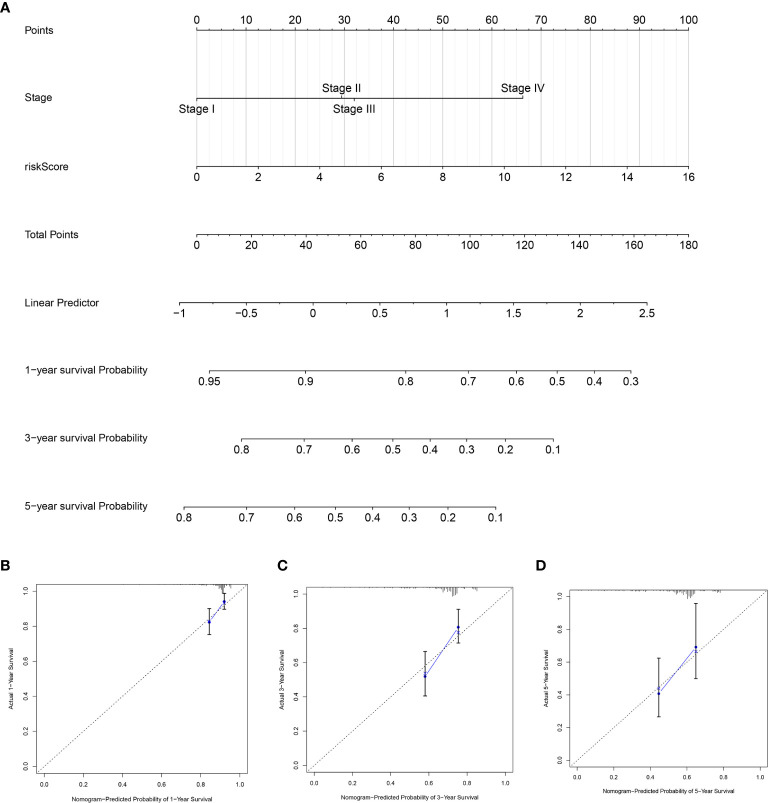
Construction of nomogram to predict the prognostic ability for the DFS in the complete dataset based on the independent factors. **(A)** A nomogram was constructed based on the independent prognosis factors to predict the prognosis of GC. The calibration plots for 1- **(B)**, 3- **(C)** and 5-years **(D)** in the complete dataset.

To further establish an individualized prediction model in the OS and DFS, we further constructed a nomogram for GC patients based on the independent prognostic factors, respectively (**Figures 9A**, **10A**). The calibration plots show that the performance of the nomogram have a good concordance with the prediction of 1-, 3-, and 5-year OS (**Figures 9B–D**) and DFS (**Figures 10B–D**).

### Identification of Candidate Drugs Targeting the lncRNA Model

**Figure 11:**
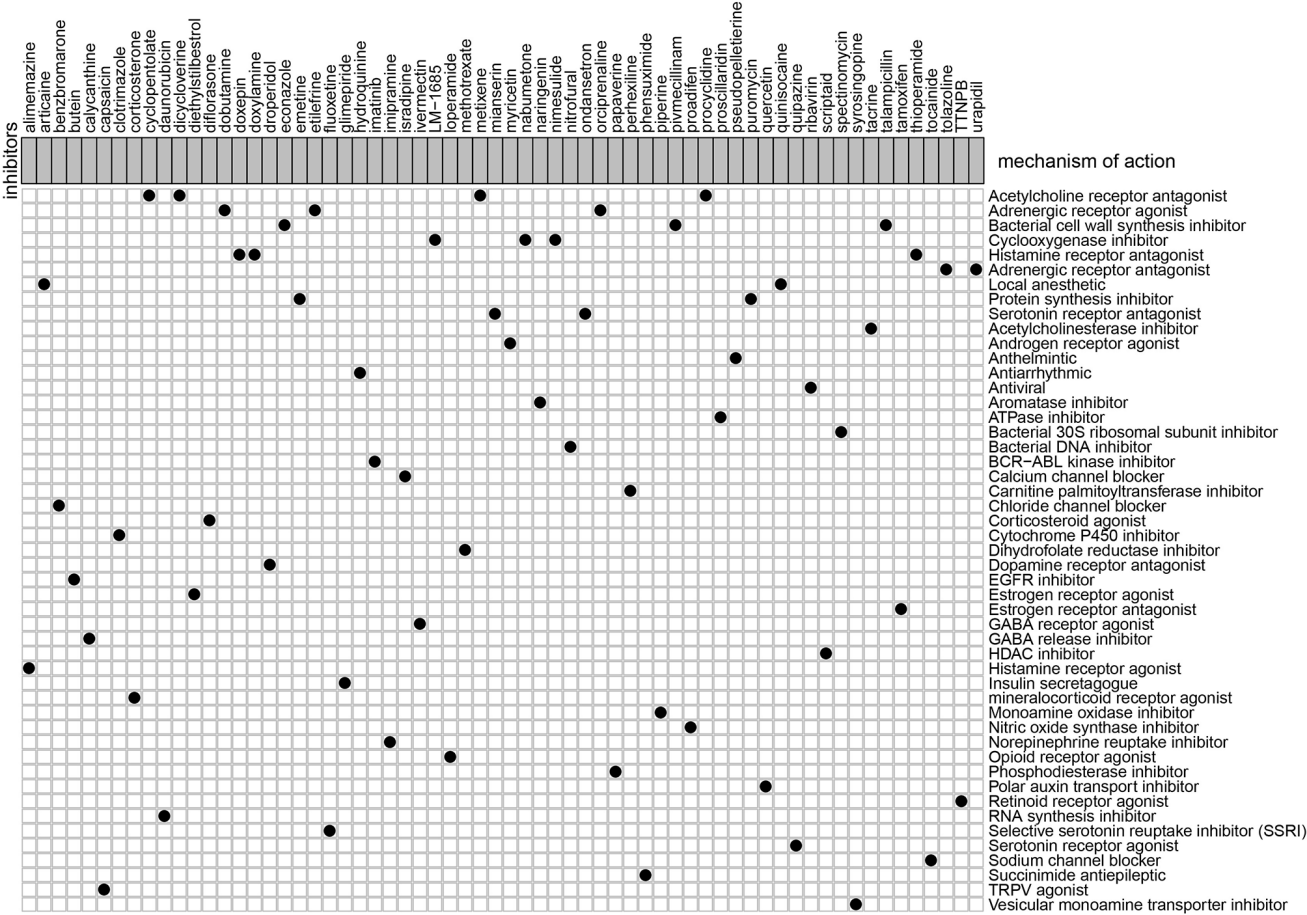
Candidate drugs that were identified by the CMAP database were targeting the lncRNA-related signature.

To explore the potential molecular drugs of GC, we first performed a differentially expression analysis between the high-risk and low risk group. As a result, a total of 150 DEGs were identified and further uploaded to the CMAP drug database. In total, 64 drugs with 45 mechanisms of action (MOA) were shared by the above drugs (**Figure 11** and **Table S2**). According to the drug result, metixene, dicycloverine, cyclopentolate, and procyclidine shared the mechanism of Acetylcholine receptor antagonist; dobutamine, orciprenaline, and etilefrine shared the mechanism of Adrenergic receptor agonist; nimesulide, nabumetone, and LM-1685 shared the mechanism of cyclooxygenase inhibitor; thioperamide, doxepin, and doxylamine shared the mechanism of Histamine receptor antagonist. Our study identified drugs targeting the m6A-related lncRNA signature and might provide therapeutic targets for further analysis.

### Consensus Clustering of m6A-Related lncRNAs

**Figure 12:**
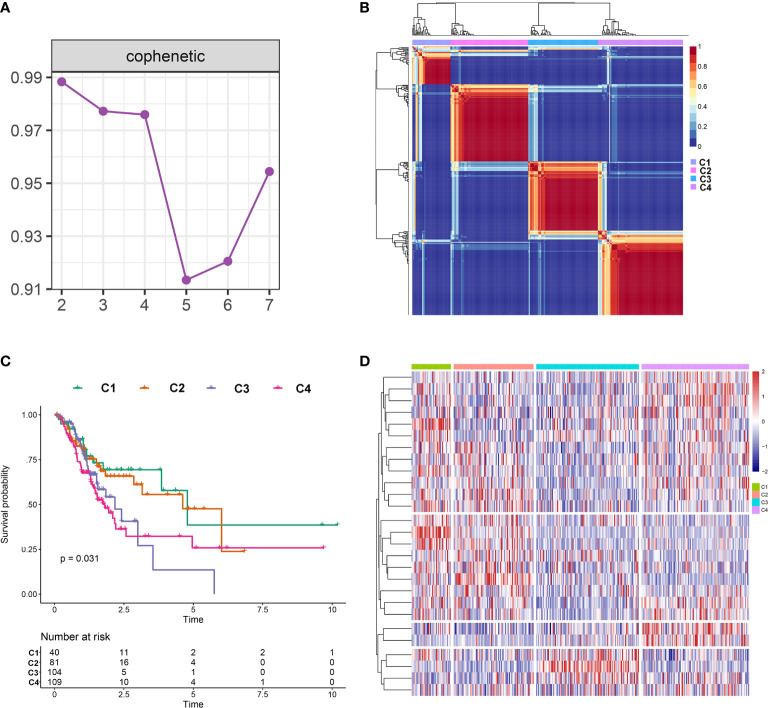
Identification of molecular subtypes in the complete dataset. **(A)** Cophenetic correlation coefficient analysis for k = 2 to k =7. **(B)** Consensus heatmap for the gene expression when k = 4. **(C)** Kaplan-Meier survival curves for the cluster when k =4. **(D)** A heatmap for the four molecular subtypes in the GC dataset.

**Figure 13:**
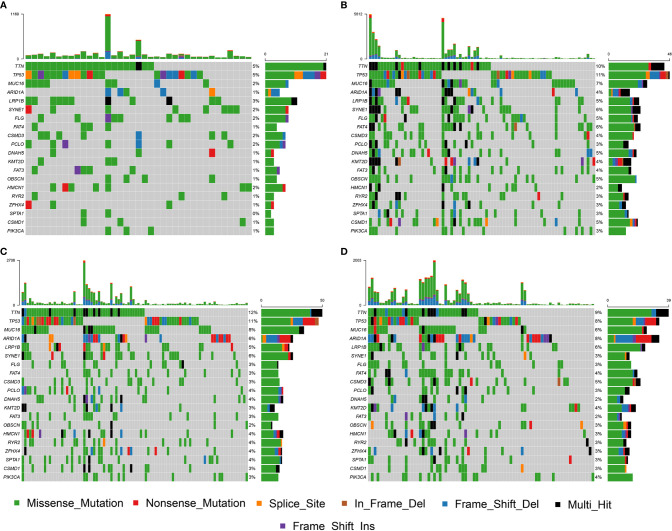
SNP alteration was identified in C1 **(A)**, C2 **(B)**, C3 **(C)**, and C4 **(D)** subtypes.

The NMF algorithm, using the m6A-related lncRNAs, was used to explore the molecular subtypes of GC. To ensure a robust and reliable subtype, low expression level lncRNAs were filtered out and retained the lncRNAs that were associated with survival of GC for univariate cox regression analysis, to ensure a robust and reliable subtype. The data was then used in a NMF clustering analysis. The cophenetic correlation coefficients were calculated to determine the optimal k value, and k = 4 was eventually selected as the optimal cutoff after comprehensive consideration (**Figure 12A**, the four subtypes were named C1, C2, C3, and C4). When k = 4, the consensus matrix heatmap still presented sharp and crisp boundaries, suggesting a stable and robust clustering for the samples (**Figure 12B**). Notably, the KM curve analysis result showed a significant survival difference among subgroups, of which C1 and C2 showed a better survival outcome compared to C3 and C4 (*P* = 0.031) (**Figure 12C**). Moreover, the different expression patterns of the lncRNAs in the four subgroups suggest that these lncRNAs might play an important role in the subgroups (**Figure 12D**). Further, we used the TIDE algorithm to predict the response of the four subtypes to immunotherapy. Subtype C1 and C2 responded better to immunotherapy compared with subtype C3 and C4 (*P* = 0.0072). In addition, we also evaluated the SNP alteration among these subtypes. We observed that subtype C3 has the highest SNP alteration, while C1 corresponded to the lowest SNP alteration (**Figure 13**).

## Discussion

Several studies have reported that tm^6^A-related lncRNAs are implicated in the development of various tumors, including GC (17). Therefore, exploring the role of lncRNAs in the prognosis or diagnosis of GC will contribute to better understanding the molecular mechanism of GC (18). However, the role of m6A-related lncRNAs in the prognosis and diagnosis of GC are still not clear and deserve further study (19).

In the present study, we systematically investigate the role of m6A-related lncRNAs in the prognosis of GC. We retrieved 339 GC patients with a survival time of more than 30 days to the downstream analyses. By performing a correlation analysis, univariate cox regression analysis, and a LASSO-penalized regression analysis, we successfully constructed a 11 m6A-related lncRNA signature. The KM curve analysis result indicated that the signature could efficiently stratify patients’ OS and has a robust prognostic value. Further ROC analysis results suggest that the lncRNA signature has a high accuracy in predicting the 5-year OS and DFS of GC. These results demonstrate that out lncRNA signature has a good prognosis and might serve as an effective biomarker for the GC.

In addition, we also evaluated the relationship between clinical factors and the lncRNA signature. We demonstrated that the lncRNA signature can independently predict the OS of GC without the need to consider other clinical variables. The GSEA result revealed that patients in the high-risk group were mainly enriched in the cytokine-cytokine receptor interaction, focal adhesion, and ECM receptor interaction pathway, and the basal transcription factors pathway was characterized by the low-risk group. Notably, the cytokine-cytokine receptor interaction pathway plays an important role in adaptive inflammatory host defenses, cell growth, differentiation, cell death, angiogenesis, and the development and repair processes aimed at restoring homeostasis (20, 21). Moreover, previous studies also demonstrated that the ECM plays an important role in cancer progression (22, 23). These results provide promising directions to clarify the underlying molecular mechanisms of the lncRNA signature of GC. Moreover, we constructed a nomogram based on the stage and lncRNA signature, after a comprehensive consideration of OS and DFS. The calibration plots showed the best performance in 1-year, 3-year, and 5-year OS, which may help in planning short‐term follow‐ups for individual treatments. Using the CMAP drug database, we identified 64 drugs with 45 mechanisms of action. These drugs include Acetylcholine receptor antagonist (metixene, dicycloverine, cyclopentolate and procyclidine), Adrenergic receptor agonist (dobutamine, orciprenaline and etilefrine), Cyclooxygenase inhibitor (nimesulide, nabumetone and LM-1685), Histamine receptor antagonist (thioperamide, doxepin and doxylamine) (24). We also identified other potential drugs that might pave the way for the implementation of targeted lncRNA-associated treatments for GC patients. In addition, according to the lncRNA expression, we also identified four robust molecular subtypes. We demonstrated that subtype C1 and C2 corresponded to a better survival outcome compared to subtype C3 and C4, and subtype C1 and C2 are more likely to respond to the immunotherapy than subtype C3 and C4. These results might provide future directions for the development of individualized treatments for GC.

Overall, our prognostic model is based on the 11 m6A-related lncRNAs, which significantly lowers the cost of sequencing and has a high clinical application value. Moreover, the prognostic model showed a good performance for survival prediction in patients with GC. Nonetheless, several limitations need to be addressed. First, our prognostic model was constructed based on the TCGA database, thus lacks a large cohort or patient cohort. Second, the expression level of lncRNAs needed to be further validated using *in-vivo* or *in-vitro* experiments.

In summary, we explored the expression levels and prognostic value of m6A-related lncRNAs through a myriad of analyses. We constructed a 11-lncRNA signature with a high prognostic value and which can serve as an independent prognostic factor for GC. To the best of our knowledge, this is the first report to develop a m6A-related lncRNA risk model for GC. The findings of this study provide new insights to understanding the role of m6A-related lncRNAs in GC and provide a basis for the development of personalized therapy.

## Data Availability Statement

The datasets generated for this study can be found in the https://portal.gdc.cancer.gov.

## Author Contributions

BM designed the study. HW and QM collected the clinical information, gene expression data, and analysis data. HW wrote the manuscript and BM provided revisions. All authors contributed to the article and approved the submitted version.

## Funding

This study was supported by the National Science Foundation of China,(Grant/Award Number: 81902383), The Doctoral Scientific Research Startup Foundation of Liaoning Province (Grant/Award Number: 2019-BS-146), the Revitalizing Liaoning Talents Program (Grant/Award Number: XLYC1907004), and the Young and Middle-aged Scientific & Technological Innovation Talent Support Plan of Shenyang City (Grant/Award Number: RC200223).

## Conflict of Interest

The authors declare that the research was conducted in the absence of any commercial or financial relationships that could be construed as a potential conflict of interest.

## Supplementary Material

Supplementary Figure 1 Landscape of 13 m6A RNA methylation regulators. **(A)** Differentially expressed analysis between tumor and normal tissue of GC among the 13 m6A RNA methylation regulators. **(B)** correlation analysis of the 13 m6A RNA methylation regulators based on the Pearson coefficient.

Supplementary Figure 2 Risk score distribution of the m6A-related lncRNA signature in the training dataset **(A)** and testing dataset **(B)**, respectively. The upper panel represents the risk score distribution from the low-risk group to the high-risk group. The middle panel represents the cases distribution, and the lower panel represents the expression of each lncRNA in the dataset.

Supplementary Figure 3 Prognostic value of the m6A-related lncRNA signature in DFS. **(A)** Kaplan-Meier curve analysis between the high-risk group and low-risk group. **(B)** The receiver operator curve (ROC) analysis for the m6A-related lncRNA signature.

Supplementary Figure 4 A comparison between the reported lncRNA model and our m6A-related lncRNA model through ROC analysis.

Supplementary Table 1 Correlation analysis between m6A-related regulator and all lncRNA.

Supplementary Table 2 Candidate drugs identified by the CMAP database.

## Abbreviations

AUC, Area under the curve; CMAP, Connective Map; DEG, Differentially expression genes; DFS, Disease free survival; GC, Gastric cancer; GSEA, Gene set enrichment analysis; KM, Kaplan-Meier; LASSO, Least absolute shrinkage and selection operator; M^6^A, N6-methyladenosine; NMF, Non-negative matrix factorization; OS, Overall survival; ROC, Receiver operating characteristic; TCGA, The Cancer Genome Atlas.
