# Supplementary figures and images for: Characterization of the Prognostic m6A-Related lncRNA Signature in Gastric Cancer
Source: Front Oncol. 2021 Apr 13;11:630260. doi: 10.3389/fonc.2021.630260 (PMC8076577; doi:10.3389/fonc.2021.630260)

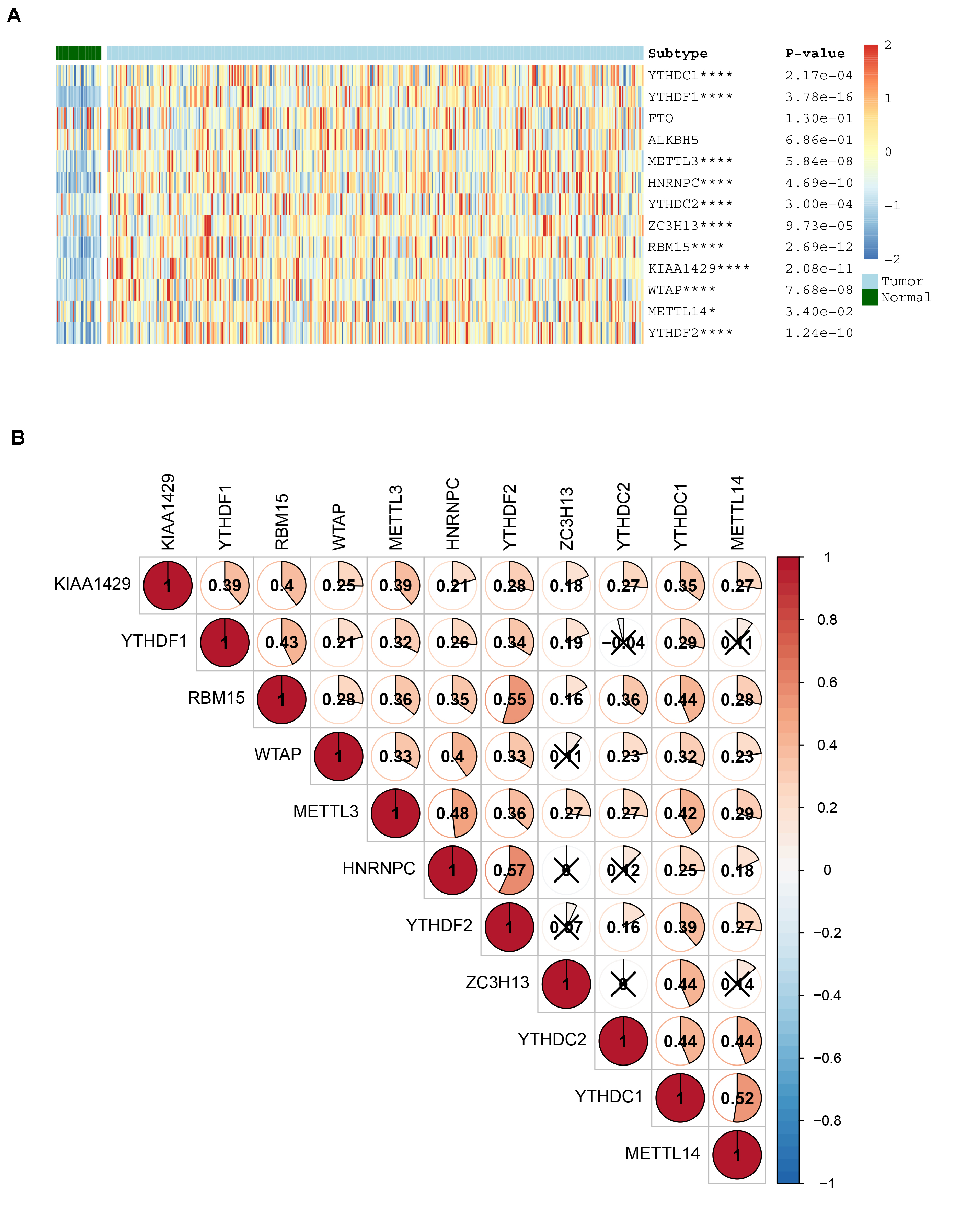

Supplement: Supplementary Figure 1 — Landscape of 13 m6A RNA methylation regulators. (A) Differentially expressed analysis between tumor and normal tissue of GC among the 13 m6A RNA methylation regulators. (B) correlation analysis of the 13 m6A RNA methylation regulators based on the Pearson coefficient. [file Image_1.jpeg]

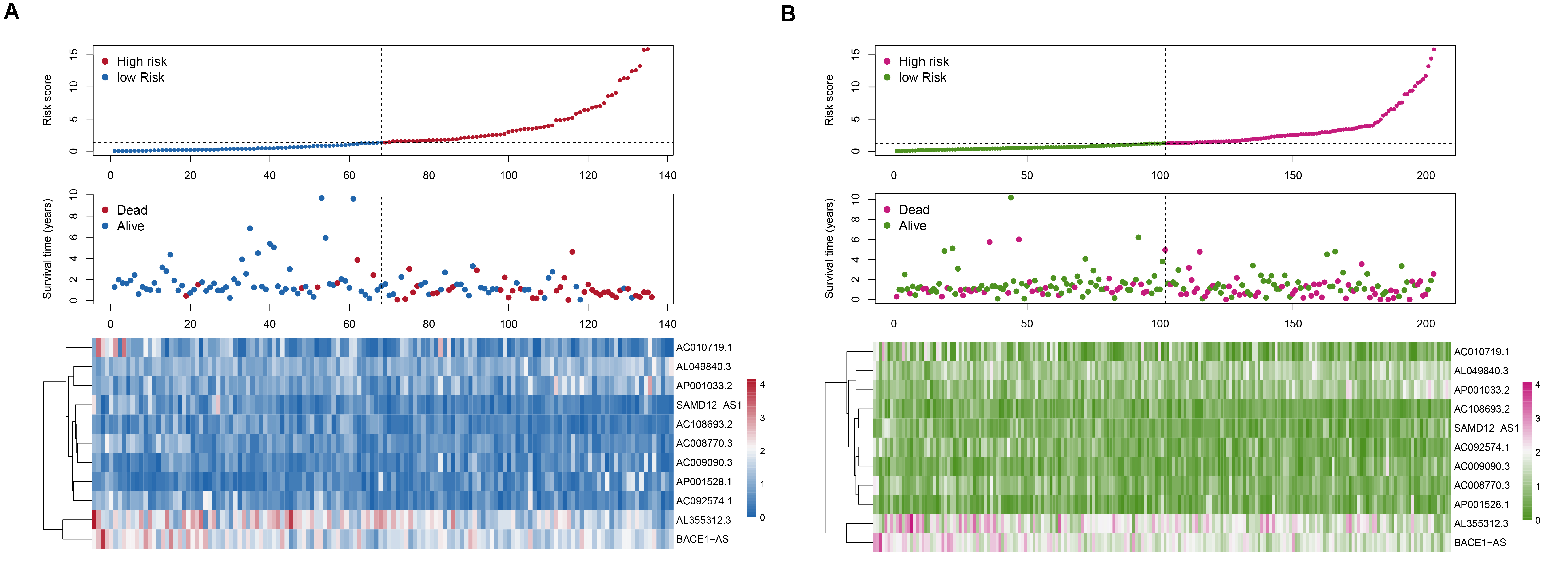

Supplement: Supplementary Figure 2 — Risk score distribution of the m6A-related lncRNA signature in the training dataset (A) and testing dataset (B), respectively. The upper panel represents the risk score distribution from the low-risk group to the high-risk group. The middle panel represents the cases distribution, and the lower panel represents the expression of each lncRNA in the dataset. [file Image_2.jpeg]

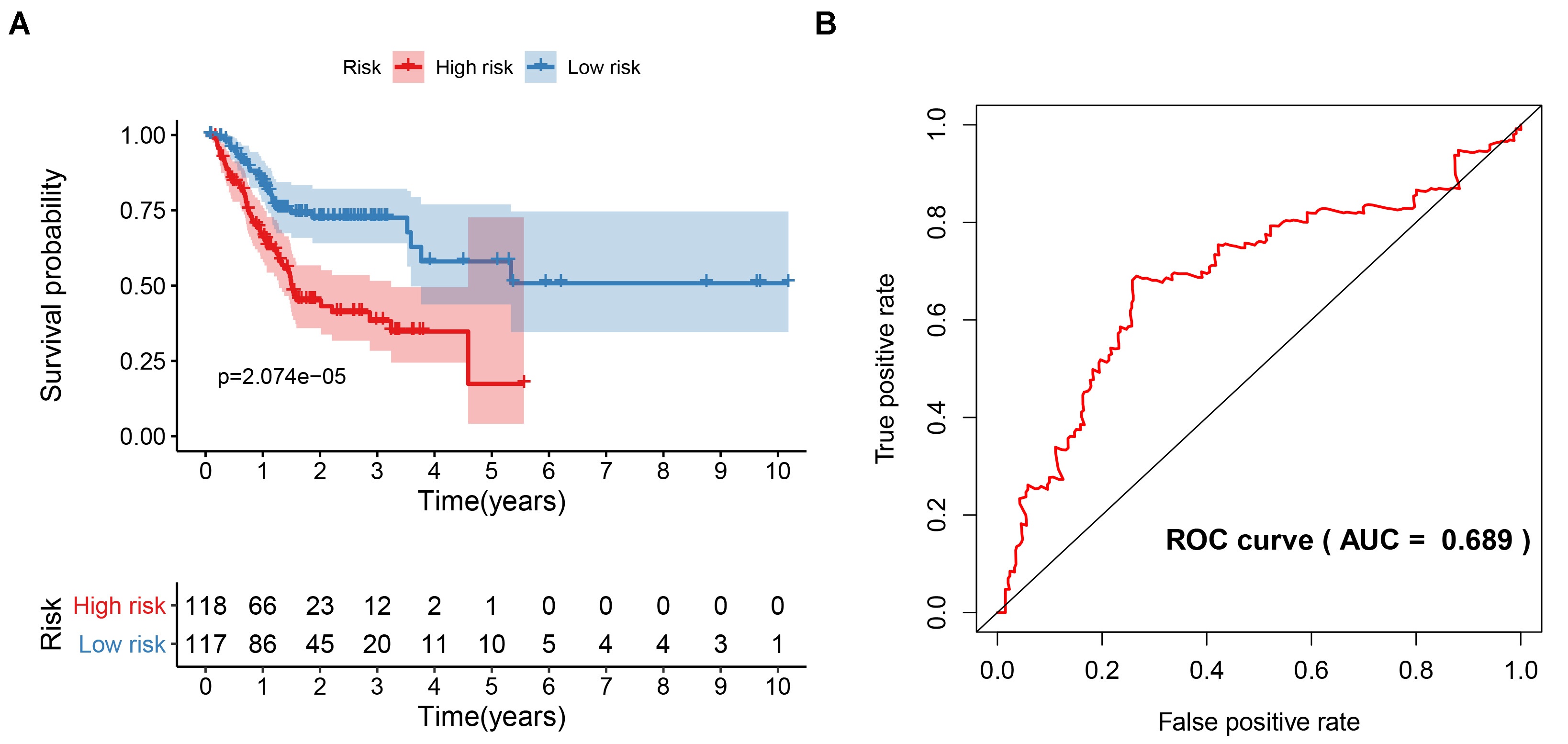

Supplement: Supplementary Figure 3 — Prognostic value of the m6A-related lncRNA signature in DFS. (A) Kaplan-Meier curve analysis between the high-risk group and low-risk group. (B) The receiver operator curve (ROC) analysis for the m6A-related lncRNA signature. [file Image_3.jpeg]

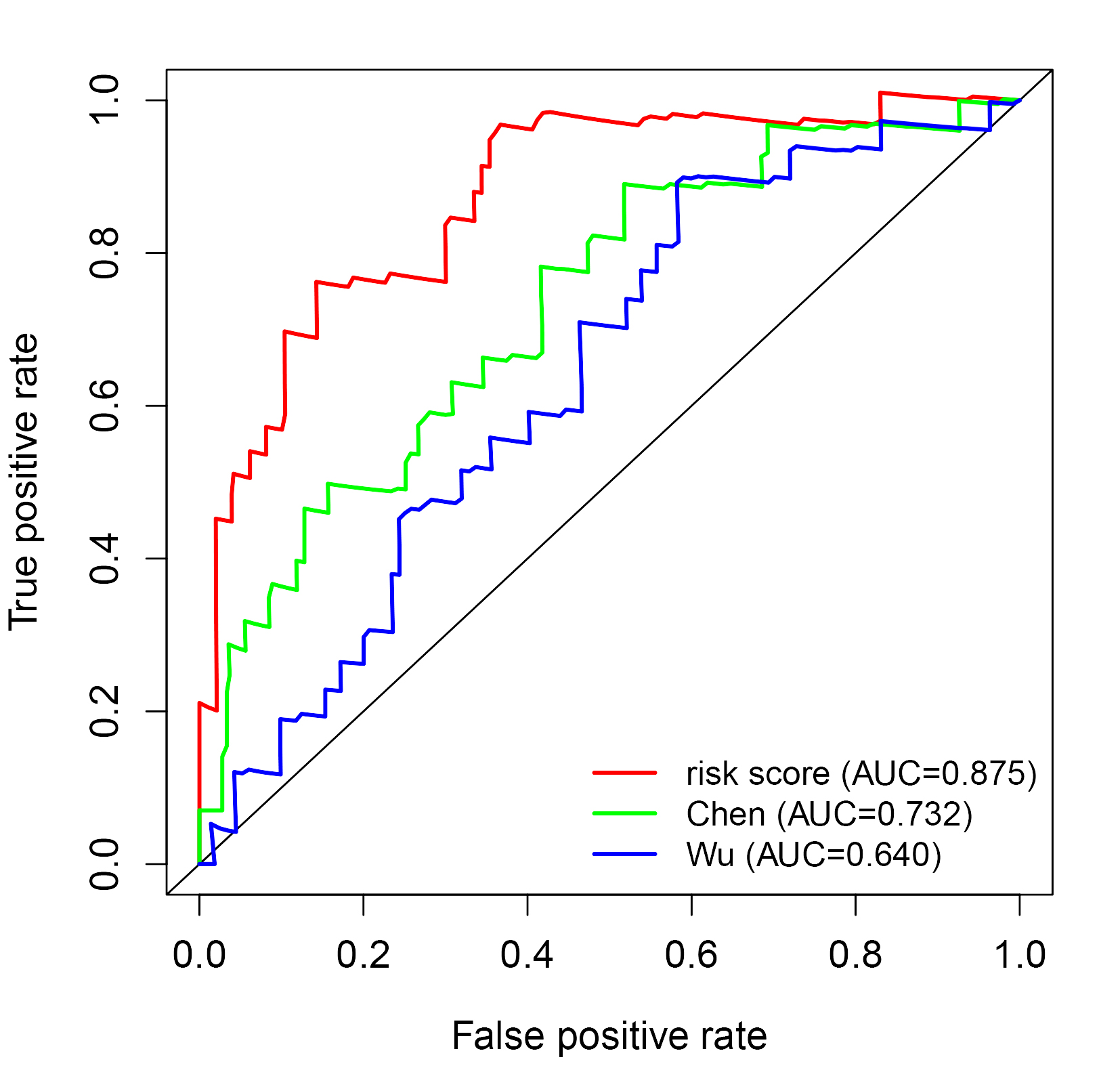

Supplement: Supplementary Figure 4 — A comparison between the reported lncRNA model and our m6A-related lncRNA model through ROC analysis. [file Image_4.jpeg]
